# Plastic responses of males and females interact to determine mating behavior

**DOI:** 10.1111/evo.14568

**Published:** 2022-08-12

**Authors:** Emily K. Fowler, Stewart Leigh, Amanda Bretman, Tracey Chapman

**Affiliations:** ^1^ School of Biological Sciences University of East Anglia Norwich NR4 7TJ United Kingdom; ^2^ School of Biology, Faculty of Biological Sciences University of Leeds Leeds LS2 9JT United Kingdom

**Keywords:** Cues, *Drosophila*, mating duration, oviposition, phenotypic plasticity

## Abstract

Individuals can respond plastically to variation in their social environment. However, each sex may respond to different cues and contrasting aspects of competition. Theory suggests that the plastic phenotype expressed by one sex can influence evolutionary dynamics in the other, and that plasticity simultaneously expressed by both sexes can exert sex‐specific effects on fitness. However, data are needed to test this theory base. Here, we examined whether the simultaneous expression of adaptive plasticity by both sexes of *Drosophila melanogaster* fruit flies in response to their respective social environments interacts to determine the value of key reproductive traits (mating latency, duration, and fecundity). To vary social environments, males were kept alone, or with same sex rivals, and females were kept alone, in same‐sex, or mixed‐sex groups. Matings were then conducted between individuals from all of these five social treatments in all combinations, and the resulting reproductive traits measured in both “choice” and “no‐choice” assays. Mating latency was determined by an interaction between the plastic responses of both sexes to their social environments. Interestingly, the mating latency response occurred in opposing directions in the different assays. In females exposed to same‐sex social treatments, mating latency was more rapid with rival treatment males in the choice assays, but slower with those same males in no‐choice assays. In contrast, mating duration was determined purely by responses of males to their social environments, and fecundity purely by responses of females. Collectively, the results show that plastic responses represent an important and novel facet of sexual interactions.

Phenotypic plasticity is the ability of individuals with a given genotype to express different phenotypes in response to biotic or abiotic variation in the environment (Komers 1997; West‐Eberhard 2003; Fordyce 2006; Dingemanse and Wolf 2013). It has been described in diverse organisms from prokaryotes to plants and animals (West‐Eberhard 2003; Dingemanse and Wolf 2013). Phenotypic plasticity results in variation in the expression of many traits affecting fitness and can represent a significant force for driving evolutionary change (Moore et al. 1997; McAdam et al. 2014; Bailey et al. 2018; Pfennig 2021). Although many studies have identified plastically responsive traits, and in some cases demonstrated that those responses are potentially adaptive (Bretman et al. 2009), the ultimate outcome of interactions between the plastic responses made by different individuals has hardly yet been tested. In dioecious organisms, for example, the interaction between the plasticity expressed by males and females in response to their social environments is expected to be an important determinant of overall fitness (Edward et al. 2014; Dore et al. 2021). A range of fitness outcomes is possible, depending upon whether the effects of plasticity expressed by one sex amplify those of the other, or cancel them out (McGhee et al. 2013; Yamaguchi and Iwasa 2015; McLeod and Day 2017; Day and McLeod 2018). Such interactions could be especially important when individuals of both sexes are making rapid and flexible responses to their social environments (Bretman et al. 2012; Bailey et al. 2018). Hence, behavioral traits are expected to be particularly sensitive to interacting effects of plasticity (Holland and Rice 1998; Bailey et al. 2018). In this study, we aimed to fill key gaps in our knowledge of social plasticity by testing (i) whether the phenotypic plasticity expressed simultaneously by both sexes interacts to influence fitness‐related reproductive traits, and (ii) whether different reproductive traits would respond differently to such interactions.

Two areas of theoretical research are relevant. First, indirect genetic effects theory describes how the effects of genes in one sex can alter the trajectory of evolution in the individuals with which they interact (Moore et al. 1997; McAdam et al. 2014; Bailey et al. 2018). It also identifies mechanisms to quantify those interactions (e.g., McGlothlin and Brodie 2009; Edward et al. 2014). This body of work reveals many instances in which the effects of genes in one sex alter the behavior or fitness of interactants (Bailey et al. 2018). Due to their flexible and dynamic nature, behavioral phenotypes may be especially sensitive (Bailey et al. 2018). A second approach has been to predict the likely outcomes of plasticity expressed in both sexes, under sexual conflict. In this, the plasticity expressed by one sex may be selected because it minimizes the potentially deleterious effects of that expressed by the other. However, the predictions arising from this body of theory vary. Interacting plasticity is predicted to either maximize the sex‐specific fitness of both sexes (McGhee et al. 2013) or of one sex at the expense of the other (Yamaguchi and Iwasa 2015; McLeod and Day 2017; Day and McLeod 2018). Variation in the predicted outcomes is determined by the strength of sex‐specific selection and whether one sex has the “power” to enforce its evolutionary interests over the other (Chapman 2006). These contrasting strands of theory have been brought together in an investigation of the effects of indirect genetic effects within sexual conflict (Moore and Pizzari 2005). This supported the idea that social selection and behavior, in particular (Holland and Rice 1998), can be important determinants of the trajectory of evolution in each sex. We conclude that empirical data on the interacting effects of plasticity, which we target in this study, are sorely needed.

Phenotypic plasticity can occur in response to variation in both the biotic or abiotic environment. Our focus here is on the biotic, and in particular on the increasing realization that how individuals alter their behavior in response to conspecifics of the same or opposite sex can be adaptive, and thus a key component of fitness (Bretman et al. 2009, 2011). Indeed, it is observed that the sociosexual environment is an important stimulus for the expression of behavioral plasticity across many different organisms (e.g., Dukas 2005; Petfield et al. 2005; Kent et al. 2008; Sarin and Dukas 2009; Davis et al. 2011; Bailey and Zuk 2012; Billeter et al. 2012; Oliveira 2012; Han and Brooks 2014; Dorset et al. 2017; Oku and van den Beuken 2017). For example, variation in social experience can influence shoaling preferences in *Gasterosteus* sticklebacks (e.g., Kozak and Boughman 2008). Exposure of males to varying social environments, or female genotypes, can alter pheromonal profiles in *Drosophila melanogaster* (Kent et al. 2008) and *Drosophila serrata* (Petfield et al. 2005), respectively. In the field cricket *Teleogryllus oceanicus*, the previous auditory environment of males affects the extent of female mate choice (Bailey and Zuk 2012).

Fruit flies have proved a tractable and valuable system for studies of social plasticity in both sexes. In the majority of investigations of plasticity to date, the focus has tended to be on only one sex at a time (often the male, in the context of intrasexual competition; Bretman et al. 2011). *Drosophila melanogaster* males are observed to respond to their intrasexual environment, and thus perceived level of sperm competition, by altering their reproductive investment, both in terms of their behavior and physiology. Specifically, males exposed to same‐sex rivals prior to mating extend mating duration and transfer into females more of two key seminal fluid proteins (SFPs)—ovulin and sex peptide (SP) (Wigby et al. 2009). Ovulin and SP induce important postmating behavioral and physiological changes to females, including increased fecundity and decreased sexual receptivity (Chapman et al. 1993; Herndon and Wolfner 1995; Heifetz et al. 2000; Liu and Kubli 2003; Wigby et al. 2009). These plastic responses benefit males, as it means that they invest in energetically costly SFPs that increase fecundity and decrease female remating, receptivity, only when it is necessary (Wigby et al. 2009). However, as the receipt of SFPs can be costly for females (Chapman et al. 1995; Wigby and Chapman 2005), the plastic responses of males to their social environments have the potential to exacerbate sexual conflict (Sirot et al. 2015). For example, females kept with socially “responding” males are reported to experience elevated death rates and higher early fecundity (Filice et al. 2020).

Studies of the responses of females to their social environments are becoming more frequent (Churchill et al. 2021; Fowler et al. 2021). For example, female fruit flies can observe and learn oviposition strategies from other females, choosing to lay eggs on a potentially “good” food substrate (Sarin and Dukas 2009), can alter their egg laying behavior in response to a male‐derived pheromone (Wertheim et al. 2006), and can exhibit variation in fecundity according to the genetic diversity of the males in their social environment (Billeter et al. 2012). Females exposed to other females prior to mating also increase their latency to start mating, and lay fewer eggs over the following 24‐h period (Churchill et al. 2021; Fowler et al. 2021). In contrast to the situation for males, the fitness benefits of these plastic responses expressed by females are not yet fully resolved. In addition, it is not yet known whether the plastic responses of females to their social environments can ameliorate the potentially costly effects arising from the expression of a male's plasticity. Hence, the ultimate effect upon reproductive traits of plasticity expressed by both sexes is likely to be important, but is not yet clear.

In this study, we used the fruit fly *D. melanogaster* to conduct tests of the effects of plasticity expressed by both sexes in response to their respective social and sexual environments on key fitness traits. Mating latency served as the reproductive trait primarily determined by immediate behavioral decisions and mating duration and fecundity by male and female physiological processes. Our two predictions were that (i) the phenotypic plasticity expressed simultaneously by both sexes would interact directly to influence important fitness‐related reproductive traits, and (ii) behavioral traits would be particularly sensitive to these interacting effects.

## Methods

### RATIONALE FOR EXPERIMENTAL DESIGN

We first varied the premating social environments of both females and males. Focal females were housed either on their own (“alone”), with three other females (“same‐sex”), or with three males (“mixed‐sex”). Males were housed on their own (“no‐rival”) or with three other males (“rival”). The aim was to manipulate social cues in each sex in a manner known to signal variation in competition and induce predictable plastic phenotypic responses whose interacting effects we could test. For focal females, we knew that manipulating exposure to other females or to males would predictably alter their fecundity (Fowler et al. 2021; Table 1). For males, we manipulated exposure to other males to predictably alter mating duration (Bretman et al. 2013; Table 1). We then mated focal females and focal males from each of the social environment treatments together in all combinations, to test for interacting effects of the plastic responses of both sexes to those environments, on mating latency, mating duration, and fecundity. Our rationale was that, if there are clear plastic responses made by each sex that increase fitness within the social environment in which they are expressed (Table 1), then we should be able to detect how often interactions override these adaptive responses, and which of the sexes suffers most from any such interacting effects.

**Table 1:** Description of the social treatments, the conditions they are likely to create and their expected effects are described

Social Manipulation Treatment		Conditions Created by the Social Manipulation Treatment	Expected Effect of the Social Manipulation Treatment on Traits Measured
Females: premating	Alone	Absence of any other individuals prior to mating signals a potential lack of mates and absence of competition for oviposition or mates.	Mating latency: no consistent effect (Fowler et al. 2021) *Expectation*: Same sex = Mixed sex = Alone Mating duration: mostly under male control (Bretman et al. 2013) *Expectation*: Same sex = Mixed sex = Alone Fecundity: low if high competition for oviposition sites (Fowler et al. 2021) *Expectation*: Same sex < Mixed sex < Alone
	Same‐sex	Presence of same sex individuals prior to mating signals that competition for oviposition sites, or potentially males, is likely.	
	Mixed‐sex	Presence of opposite sex individuals prior to mating signals that females may have the ability to express direct mate choice and are likely to experience the effects of competitions between males for matings and fertilizations.	
Males: premating	No‐rival	Absence of same sex rivals prior to mating signals low competition for matings and fertilizations, and a potentially reduced chance of mating overall.	Mating latency: potentially shorter if male‐male competition high (Bretman et al. 2009) *Expectation*: Rival < No rival Mating duration: short if male‐male competition low (Bretman et al. 2009) *Expectation*: No rival < Rival Fecundity: low if male‐male competition low (Bretman et al. 2009) *Expectation*: No rival < Rival
	Rival	Presence of same sex rivals prior to mating signals that competition for matings and fertilizations is likely.	
Mating arena assay: one or two males present	Choice	Presence of two males in the mating arena allows females to simultaneously assess different males. Males can also directly compete. Assessments of competition can be based on previous and current experience, by both sexes.	Mating latency: shorter if male‐male competition possible (Bretman et al. 2009) *Expectation*: Rival < No rival Mating duration: shorter if male‐male contests possible (Bretman et al. 2009) *Expectation*: Rival < No rival Fecundity: no immediate response (Bretman et al. 2009) *Expectation*: Rival = No rival
	No choice	Presence of only one male in the mating arena offers no opportunity for direct comparisons between males. Assessment of competition, and choice of whether to mate at all, is indirect and based upon previous experience only.	

There is a rich literature on how to test for mating‐related traits (Coyne et al. 2005; Dougherty and Shuker 2014). Mate preferences appear generally stronger under designs that allow choice, potentially due to higher costs of mate rejection under “no‐choice” paradigms (Dougherty and Shuker 2014). We had predicted that focal females might respond to a male carrying a more costly ejaculate (i.e., males that have been exposed to a high level of sperm competition before mating) by avoiding mating with them altogether. However, such effects might be less strong under “no‐choice” tests. So, to minimize any confounds due to testing methods, we examined if focal males from the rival and no‐rival social environments differed in their ability to secure matings under both “choice” and “no‐choice” scenarios (Dougherty and Shuker 2014). The choice assays introduced the effect of direct competition between males of different environments and of direct comparison of those males by females (Table 1). This also allowed us to test for mating success of rival and no‐rival males with females of each of the social environments. In the no‐choice setup, the effects of direct competition/comparison between males were removed (Table 1).

### DETAILED METHODS

#### Stock maintenance and fly collection

Wild‐type *D. melanogaster* flies were from a large laboratory population originally collected in the 1970s in Dahomey (Benin). Flies were maintained in stock cages with overlapping generations on SYA medium (Sugar Yeast Agar: 100 g brewer's yeast, 50 g sugar, 15 g agar, 30 mL Nipagin [10% w/v solution], and 3 mL propionic acid, per liter of medium). SYA medium was used throughout the experiments and all flies were cultured and reared, and all experiments performed, at 25°C, 50% relative humidity, on a 12:12 h light:dark cycle. Eggs for all experimental manipulations were collected from population cages using grape juice agar egg collection plates (275 mL H_2_O, 12.5 g agar, 250 mL red grape juice, 10.5 mL 10% w/v Nipagin solution) supplemented with live yeast paste. First instar larvae were picked into glass vials (75 × 25 mm) containing 7 mL SYA medium at a density of 100 larvae per vial. Adults were collected within 8 h of eclosion, separated into same sex groups using ice anesthesia, and stored 10 per vial. Adults were stored under these conditions for 4 days and allowed to reach sexual maturity until use in the experiments.

#### Manipulation of focal female and male premating social and sexual environments

##### Females

**Figure 1:**
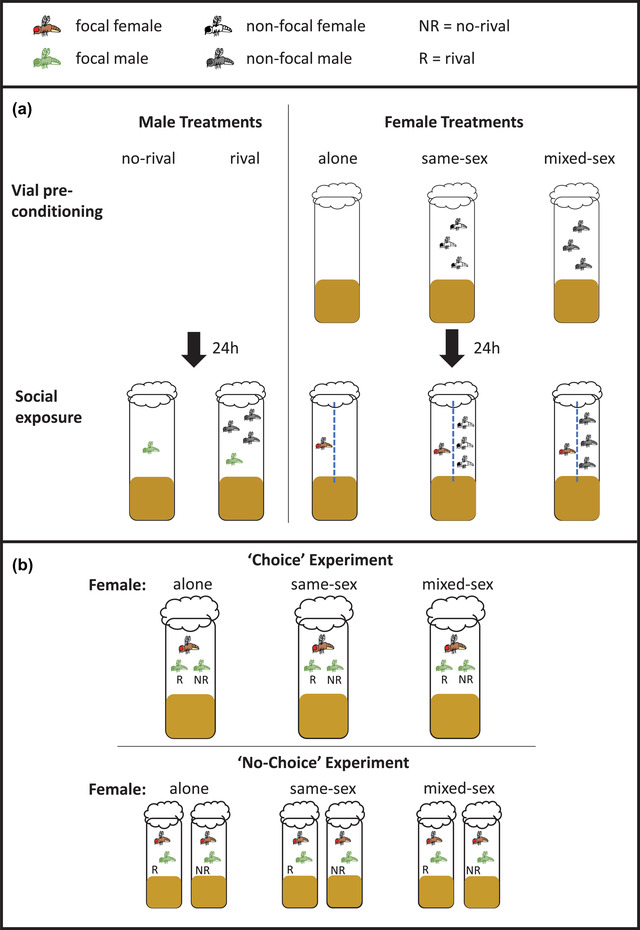
Pre‐ and postmating environment manipulations. (a) Premating social environment manipulations. For the female social treatments, nonfocal flies were placed into vials 24 h before the introduction of the focal female to transfer the respective residual social cues (“same‐sex” and “mixed‐sex” treatments [vial preconditioning]). After 24 h, the preconditioned “same‐sex,” “mixed‐sex,” and “alone” treatment vials were divided using perforated acetate sheets (blue dashed line) to separate focal females from the nonfocal flies. (b) Mating assay setup for the choice and no‐choice experiments. Vials in the choice experiment consisted of a single female from one of the three social treatments in a vial with both a rival (R) and no‐rival (NR) male. The no‐choice experiment consisted of a single female from one of the three social treatments in a vial with either a rival or no‐rival male.

The three female social environment treatments were as follows: alone, same‐sex, and mixed‐sex. Flies were collected and stored as described above, and focal females were randomly allocated to one of the three social environments when they were 4‐day old. To set up the social environments, flies were anesthetized under light CO_2_ and brushed into the social arenas (glass vials containing 7 mL food medium). To prevent the focal female from mating with the nonfocal males in the mixed‐sex treatment (and thus confounding the different social exposures), focal females in all treatments were placed on one side of a perforated acetate divider in the center of each vial (Fig. 1). The focal female was placed one side of the divide and the nonfocal flies (three females for the same‐sex treatment; three males for the mixed‐sex), or no flies (alone treatment), on the other. This setup allowed the transmission of auditory, olfactory, and visual cues but prevented physical contact between the focal and nonfocal flies. Females may require direct contact with cues deposited on the food surface by cohabitants to express socially mediated plasticity (Fowler et al. 2021). Therefore, we preconditioned social arenas prior to the introduction of the focal females, by allowing nonfocal flies in the mixed‐sex and same‐sex treatments access to the entire vial for 24 h prior to adding the acetate dividers and focal female (Fig. 1a).

##### Males

The two male social environment treatments were rival and no‐rival. The rival treatment consisted of one focal male housed with three nonfocal males, and the no‐rival treatment was one focal male housed alone. Males were collected and stored as described above. To distinguish them from focal males in the rival treatment, nonfocal males were wing‐clipped under CO_2_ anesthesia prior to setting up the social treatments. Wing‐clipping nonfocal males does not affect the known plastic responses of focal males to rivals (i.e., increased mating duration and increased mate fecundity; Bretman et al. 2011). When males were 4‐day old, focal males were randomly assigned to either the rival or no‐rival treatment. Social arenas again consisted of glass vials containing 7 mL food medium. Because there was no mixed‐sex treatment for the focal males, there was no requirement to use dividers to prevent matings during the social exposure phase. This also meant that male social treatments also did not require vial preconditioning (Fig. 1a).

All females and males were kept in the above premating social environments for 48 h (Fig. 1a). We then conducted fully factorial mating trials between the focal males and focal females in two separate experiments. In the first, the mating test was conducted using a “choice” design, whereby each focal female was exposed to two males in the mating arena (one male from each of the rival or no‐rival social treatments). In the second, mating tests consisted of one female and one male in a “no‐choice” design (Table 1).

### EFFECT OF MALE AND FEMALE SOCIAL ENVIRONMENT ON MATING LATENCY, DURATION, AND FECUNDITY UNDER CHOICE CONDITIONS

In the choice experiment, each focal female was placed in a mating arena with two focal males, one from the rival and one from the no‐rival treatment (Fig. 1b). To allow identification of rival or no‐rival males in the mating arena, focal males had been wing‐marked with a dot of either red or black ink (Staedtler Lumocolor marker) under CO_2_ anesthesia prior to setting up the social treatments. Wing marking was balanced across rival and no‐rival treatments to ensure that any effect on female choice would not introduce directional bias. On the day of the mating experiment, each focal female and two marked focal males were aspirated into a vial and given 90 min to start mating. Mating latency and mating duration were recorded as was the ink color of the male that was chosen/secured the mating. Females were only allowed to mate once, and immediately following the end of copulation both males were removed and females were left in the mating vials for 24 h to lay eggs. Females were then discarded and the number of eggs laid was recorded. We conducted mating trials with a total of 131 focal females: 46 from the female alone treatment, 42 from the same‐sex treatment, and 43 from the mixed‐sex treatment (Table S1).

### EFFECT OF MALE AND FEMALE SOCIAL ENVIRONMENT ON MATING LATENCY, DURATION, AND FECUNDITY UNDER NO‐CHOICE CONDITIONS

In the no‐choice experiment, each focal female was paired with a single focal male (rival or no‐rival) in the mating arena. On the day of the mating experiment, each focal female and focal male were aspirated into a vial (Fig. 1b). Pairs were given 90 min to start mating, and mating latency and duration were recorded. Immediately following the end of copulation, the male was removed and the female left to lay eggs for 24 h. Postmating egg data were collected as above. In total, 266 “no‐choice” trials were set up (Table S1).

### STATISTICAL ANALYSES

All analyses were carried out in R version 4.0.5 (R Core Team 2021) using the stats and survival packages (version 3.2.10; Therneau 2021). Post hoc tests were conducted using emmeans (version 1.6.2‐1; Lenth 2021). In each case, we first fitted a full model including male and female treatment and their interaction. If there was no evidence for a significant interaction, we removed the term and compared model fits using anova() from the stats package, specifying a Chisq test for binomial and cox models and an *F* test for quasipoisson models. If there was no significant difference between the complex and simple models, we accepted the simpler model. We repeated this model reduction for nonsignificant main effects. To generate the final *P*‐values reported in the results section, we ran anova() on all final models using the appropriate test as specified above.

#### Mating latency

Cox proportional hazards models from the survival package were used to analyze mating latency. Male and female social treatment and their interaction were included in the final models as fixed factors in both the choice and no‐choice assays.

#### Mating duration

We used mating duration as the response variable in a linear model. For both the choice and no‐choice assays, we initially included both male and female environment, plus their interaction as fixed factors in the model. In both cases, we reduced these to final models containing only the main effect of male social environment.

#### Fecundity

The number of eggs laid in the 24 h after mating was used as the response variable in a generalized linear model (GLM) with a quasipoisson error distribution, to account for overdispersion. For both choice and no‐choice assays, we initially included male and female environment plus their interaction as fixed factors. For the choice data, we reduced this to a final model containing only female social environment as the main effect. Post hoc tests were then conducted using the emmeans package. For the no‐choice data, the final model included both male and female social environment but not their interaction. We also analyzed fecundity data a second way, by subsetting by female social environment and testing the effect of male treatment within each subset. In each of these models, egg count was the response variable in a GLM with quasipoisson errors and male social environment was a fixed factor.

#### Mating success in the choice assay

In the choice assay, we were also able to test for differences in mating success of rival and no‐rival treatment males paired with the females from each of the female social treatments. First, to determine if either wing marker color or male treatment had an effect on the outcome of the mate choice assay, we used two separate Pearson's Chi‐squared tests with Yates’ continuity correction. Then to test how female social environment affected the outcome of the mate choice assay, we used the proportion of total matings secured by the rival treatment males as the response variable in a GLM with a binomial error distribution. Female social environment was specified as a fixed factor. The emmeans package was used to predict proportions based on this model and derive 95% confidence intervals.

## Results

### EFFECT OF MALE AND FEMALE SOCIAL ENVIRONMENT ON MATING SUCCESS, MATING LATENCY, DURATION, AND FECUNDITY UNDER CHOICE CONDITIONS

**Figure 2:**
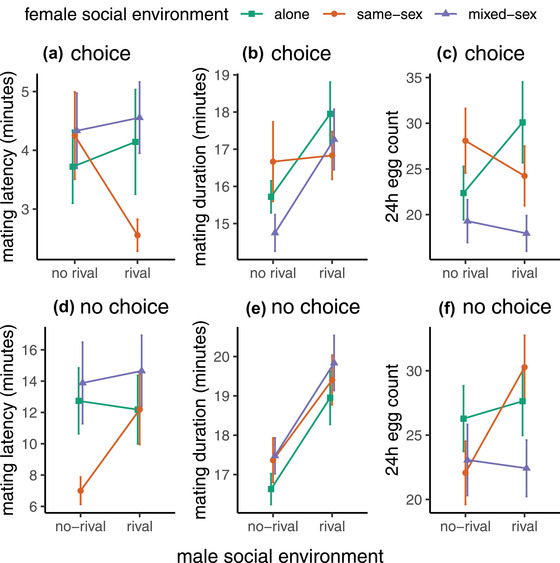
Mating latency, duration, and fecundity in males and females subjected to differing social environments. (a, d) Latency to start mating; (b, e) mating duration; and (c, f) fecundity (eggs produced during the 24 h period after mating). Females were either housed on their own (alone, green squares), with three other females (same‐sex, orange circles) or with three males (mixed‐sex, purple triangles) for 48 h prior to mating. Males were housed either on their own (no‐rival treatment) or in a group of four males (rival treatment) 48 h prior to mating. Panels (a–c) represent data generated from matings in the choice scenario, where each female was exposed to two males from different social backgrounds simultaneously. Panels (d–f) represent data from matings in the no‐choice scenario, where each female was exposed to either a rival or no‐rival male. Error bars represent the standard error. Mating latency was determined by the interaction between male and female social environments (in opposite directions in panels [a] and [d]); mating duration by the male's social environment (panels [b] and [e]), and fecundity mostly by the female's social environment, particularly under choice conditions (panels [c] and [f]).

**Figure 3:**
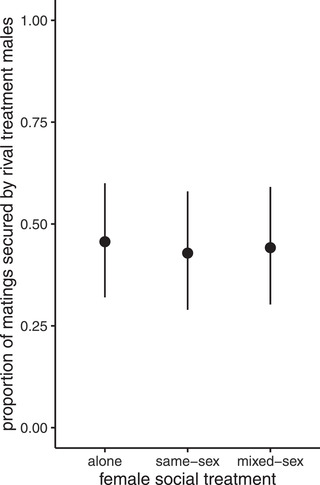
Proportion of matings secured by males from the “rival” social treatment when in direct competition with a “no‐rival” male. Males were placed with a female from one of three social environments (alone, same‐sex, and mixed‐sex); 95% confidence intervals of predicted proportions derived from least square means testing of the model are shown.

There was a significant effect of the interaction between male and female social treatment on mating latency (*χ* ^2^ = 6.52, *df* = 2, *P* = 0.038). The model coefficients show that this arose from the interaction between males and same‐sex females (Table S3). Females from the same‐sex environment on average mated with rival males faster than no‐rival males (rival = 2.56 min; no‐rival = 4.25 min), compared with alone and mixed‐sex females whose average latency with rival males was slightly longer than no‐rival males (Figs. 2a, S1; Table S1). There was no significant interaction effect of male and female social environment on mating duration (Fig. 2b; Table S3), but the main effect of male social environment was significant (*F*_(1,129)_ = 7.28, *P* = 0.008). Males that had been housed with rivals and secured a mating mated for longer than successful no‐rival males (mean duration for: rivals = 17.4 min; no‐rivals = 15.7 min). There was no significant interaction effect of male and female social environment (Fig. 2c; Table S3) on fecundity (eggs laid in the 24 h after mating) but the main effect of female social treatment was significant (*F* = 3.93, *df* = (2,127), *P* = 0.02). Post hoc testing revealed females housed alone and in same‐sex environments laid significantly more eggs than mixed‐sex females (mean egg counts: same‐sex = 26.4; alone = 25.9; mixed‐sex = 18.7). The choice test setup also allowed us to test for differences in the success or choice of the different males. However, in contrast to the behavioral trait of mating latency, there was no significant effect of plasticity expressed by either sex on mating success (male treatment: *χ* ^2^ = 2.99, *df* = 1, *P* = 0.083; female treatment: *χ* ^2^ = 0.07, *df* = 2, *P* = 0.97) (Tables S2 and S3; Fig. 3). Wing marking color also had no significant effect on male mating success within any of the female treatments (Table S4).

Overall, in these choice tests, the results showed evidence for the interacting effects of plasticity expressed by males and females, but only on mating latency. In contrast, mating duration was determined solely by the male's social environment (being longer following exposure to rivals prior to mating) and postmating fecundity was determined by female social environment (being lower in mixed‐sex females). There was no effect of plastic responses of either sex on mating success.

### EFFECT OF MALE AND FEMALE SOCIAL ENVIRONMENT ON MATING LATENCY, DURATION, AND FECUNDITY UNDER NO‐CHOICE CONDITIONS

As found in the choice tests, there was a significant effect of the interaction between male and female social treatment on mating latency (Table S5; *χ* ^2^ = 5.90, *df* = 2, *P* = 0.05). The interaction was again driven by the response of the same‐sex treatment females (Fig. 2d). However, in contrast to the choice scenario, same‐sex females in the no‐choice assay had a longer average latency to mate with rival males than no‐rival males (rival = 12.2 min; no‐rival = 7 min). Females from mixed‐sex and alone environments had similar average mating latencies with both rival and no‐rival males (Figs. 2d, S1; Table S1). There was no significant interaction effect of male and female social treatment on mating duration (Fig. 2e; Table S5), but there was again a significant main effect of male social environment (*F*_(1,253)_ = 23.01, *P* < 0.001). The mean mating duration of males previously housed with rivals was 19.4 min, compared to 17.1 min for no‐rival males. There was no significant effect of either male or female social environment, nor their interaction, on fecundity (Fig. 2f; Table S5). However, there appeared to be a trend for females from the same‐sex environment to produce more eggs following matings with rival treatment males. This was supported by separate analyses of the effect of male environment on each female treatment. Females from the same‐sex treatment produced significantly more eggs when mated to a rival male compared with a no‐rival male, but there was no significant impact of male treatment when females were housed alone or in a mixed‐sex environment (alone: *F*_(1,88)_ = 0.135, *P* = 0.714; mixed‐sex: *F*_(1,81)_ = 0.033, *P* = 0.856; same‐sex: *F*_(1,78)_ = 5.2, *P* = 0.025; Table S6; Fig. 2f).

Overall, in the no‐choice tests there was again evidence for an interacting effect of plasticity on mating latency. Interestingly, the mating latency effect was in the opposite direction, with males previously exposed to rivals being slower to mate with females from the same‐sex environment, but not other treatment females. Consistent with the choice tests, mating duration was again determined only by the male's social environment, with rival males mating for longer across all contexts. However, in contrast to the finding for the choice tests, here we found no significant effects of male or female social environment on fecundity.

## Discussion

In this study, we varied the sociosexual environment of each sex and determined, using both choice and no‐choice experimental designs, the overall effect of these potentially adaptive plastic responses to those environments on key fitness‐related reproductive traits. We tested two predictions: first, that the phenotypic plasticity expressed simultaneously by both sexes would interact directly to influence fitness‐related reproductive traits; second, that the behavioral traits tested would be particularly sensitive to these interacting effects. Both predictions were only partially upheld.

Our main finding was that the interacting responses of both sexes to their sociosexual environment did indeed influence the expression of the behavioral reproductive trait of mating latency, in line with prediction 1. Interestingly, the effects on mating latency were observed under both choice and no‐choice conditions but were manifested in opposing directions. However, not all reproductive traits were determined by interacting effects of plasticity: mating duration was determined by responses to the social environment by males, and fecundity by that of females. In line with prediction 2, the behavioral reproductive trait of mating latency was sensitive to interactions between the plastic responses of males and females and the physiological investment traits of mating duration and fecundity were not. However, an exception was the behavioral trait of mating success (measured only in the choice assay), which was relatively insensitive to plasticity. We used choice and no‐choice designs under the expectation that preferences would be stronger under choice assays (Dougherty and Shuker 2014). This expectation was not met—there were opposing responses of mating latency in the different choice assays. The results highlight that social environments can exert important influences both across time, as the previous social environment can influence present behavior, and across different contexts, as the “nonsexual” social environment can affect mating behavior. Our findings support the idea that social environments can have pervasive effects in several different dimensions of an individual's life history. We discuss the findings for each of the traits in more detail, below.

### INTERACTING EFFECTS OF PHENOTYPIC PLASTICITY IN BEHAVIORAL TRAITS: MATING LATENCY WAS DETERMINED BY THE PLASTIC RESPONSES OF BOTH SEXES

The study provided evidence for the interacting effects of the plastic responses of both sexes to their social environments on the behavioral trait of mating latency. The interacting effects were centered around differences in the responses of same‐sex treatment females. This suggests that there was something qualitatively or quantitatively distinct about the plastic responses of these females, or the way they were perceived by males, in comparison to females kept on their own or kept with males prior to mating. Given that the same‐sex females may perceive that mating opportunities are low, they may be less resistant to mating attempts than females from other treatments. The potentially lower choosiness of same‐sex females could exacerbate differences between rival and no‐rival males. Experiments manipulating the social environment of one sex alone (Table 1) highlighted shorter mating latency as an adaptive response to male‐male competition. Hence, mating latency was expected to be shorter (i) in choice conditions, which was generally the case for all female treatments, and (ii) in the +rival treatments, which occurred only in matings with choice/same sex treatment females (the opposite was true for the no choice/same sex combination). Therefore, the significant interaction between same sex and ±rival treatments had the potential to override the fitness benefits to males of short mating latencies. Although short latencies can be beneficial for males, as they gain fertilizations sooner, they may also be costly to females if associated with frequent matings. However, short mating latency could be beneficial for females if it leads to matings with high‐quality males. The overall fitness consequences of these interacting mating latency effects should be measured under a range of choice scenarios.

Variation in focal female social environments was achieved by placing perforated acetate dividers. This allowed us to generate a mixed sex treatment while preventing mating by focal females across all social environments (Fig. 1). We know that females may require direct contact with residual cues to initiate responses to social environments (Fowler et al. 2021). Therefore, we preconditioned vials (allowed the relevant nonfocal treatment flies to range freely over the whole vial) prior to the introduction of the acetate divider and focal female. Thus, focal females were provided with direct access to known treatment‐relevant cues. However, it is possible that exposure of focal individuals to as of yet unknown cues might be restricted in some way by the use of the dividers. Therefore, the magnitude of any effects of female social treatment is expected to be conservative. It is also possible that the use of the perforated dividers themselves might alter the physiochemical properties of the vials, although as all female social treatments controlled for this, it should not represent a confound.

A striking finding was the opposing direction of the response of mating latency in the no‐choice versus choice experiments. This ran counter to our general expectation that effects would be qualitatively similar across the choice paradigms, although stronger in the choice assays (Dougherty and Shuker 2014). Differences in preference across assay designs may arise due to increased costs of rejection in a no‐choice scenario (Dougherty and Shuker 2014). However, if this were the case then it would imply that only same‐sex social treatments in females experienced such costs. The observed outcomes could then arise if there were differential costs of rejection in choice and no‐choice assays for the different social treatments arising from differing expectations of mate or resource competition set up by the previous versus proximate environments. To set up the choice assays, we used wing clipping to identify nonfocal males. This does not represent a confound as focal males respond to wingless, wing‐clipped, or nonclipped rivals similarly by extending mating duration and increasing the egg laying of their mates (Bretman et al. 2009). However, wing threats are an important aspect of male‐male competition and it is possible that wing clipping nonfocals could have given the focal male a consistent competitive advantage and generated a potential winner/dominance effect. For example, if there was a consistent winner effect in the rival condition, this could potentially explain why rival treatment males mated faster in the choice setting but slower in the no choice. However, should this have been the case, then it should have been evident across all female social environment treatments rather than in only the same‐sex treatment.

Overall, the mating latency result is important as it shows that interacting responses can determine the evolutionary trajectory of this reproductive trait in a manner that may contrast with that found for others. The results suggest that the proximate mating environment as well as previous social experience both influence mating latency. It also suggests mating latency may be uncoupled from overall mate choice preference. This is because fewer rival males secured matings than no‐rivals overall in the choice experiment, even though the rival males that did mate were faster to start copulating with same‐sex females. The presence of interacting effects shows that mating latency is especially responsive to the rapid and flexible responses of each sex to their social environments (Bretman et al. 2012; Bailey et al. 2018).

### NON‐INTERACTING PHENOTYPIC PLASTICITY IN PHYSIOLOGICAL TRAITS

#### Mating duration was determined primarily by plasticity expressed by males

In contrast to the interacting effects described above, plasticity in mating duration was primarily determined by the responses of males to their social environments. In both the choice and no‐choice assays, males of the rival treatment always mated for longer than males of the no‐rival treatment. Therefore, we conclude the mating duration effect was independent of, and could not be overridden by, female social environment effects in both mating scenarios. This indicates that some reproductive phenotypes are influenced by the plasticity expressed by only one sex. The mating duration results are consistent with previous findings (Lizé et al. 2011; Price et al. 2012; Bretman et al. 2013). Under elevated sperm competition, mating duration may be correlated with an increased transfer of SFPs and sperm to the female (Bretman et al. 2009; Garbaczewska et al. 2013;, Moatt et al. 2014; Hopkins et al. 2019), although the extent to which this occurs depends upon whether males are exposed to rivals and, if so, how many they face (Hopkins et al. 2019). If there is variation in the amount of SFPs transferred by males in response to their environment that is only loosely coupled with mating duration itself, this could explain why there was no significant effect of the other social treatments on mating duration. If females do receive elevated levels of SFPs, they may experience reduced mating propensity (Mazzi et al. 2009) and increased fecundity in the short term (Bretman et al. 2009). This would be advantageous for the male, as preventing females from remating increases paternity share via elevated success in sperm competition (Bretman et al. 2009). However, these extended postmating effects may also be costly to females if re‐mating opportunities with higher quality males are lost or because receipt of SFPs is costly to females (Chapman et al. 1995). Plasticity in mating duration in response to rival exposure is primarily under male control (Bretman et al. 2013). However, *Drosophila* females can exert some influence on mating duration (Mazzi et al. 2009), and so the fact that the social environment of the female did not affect mating duration in our study suggests that the costs to males from sperm displacement are greater than costs to females from missed matings. Hence, there may be greater selection acting on males to “guard” females through increased mating duration than on females to resist longer matings.

#### Fecundity was determined primarily by plasticity expressed by females

Fecundity was primarily determined by the female's social environment, being lowest in mixed‐sex and highest in same‐sex females. This implies that the extent to which fecundity is influenced by receipt of SFPs can be significantly altered by the female's premating social environments. Female fecundity will be affected by the speed and efficiency of processing of SP and ovulin. This is partly dependent on a network of female‐expressed proteins, giving opportunity for the female to exert control over the effects of SFPs (Sirot et al. 2015). It may benefit females to precisely control the level of SFP processing in response to their own perception of the chances of re‐mating and the availability of resources such as nutrients and oviposition sites. For example, the costs of receiving SP are likely caused by a lower re‐mating rate and increased short‐term investment in egg production that may trade off against somatic investment. However, if opportunities for re‐mating are low, as might be signaled in the same‐sex treatment when females are exposed to other females, then increased short‐term fecundity mediated through SFPs may benefit females, or at least be costly to resist. Differences in the speed of processing of seminal fluid components such as SP by females might be a reason why, although we might then have expected higher fecundity in females exposed to rival males (which mate for longer and transfer more SP [Wigby et al. 2009]), which was not observed.

### NON‐INTERACTING PHENOTYPIC PLASTICITY: MATING SUCCESS IN THE CHOICE ASSAYS

In each of the three female social environment treatments within the choice experiment, no‐rival males consistently secured more matings than did rival males, although this effect was not significant. The potentially higher mating success of no‐rival males over rival males could be explained by female preference or male competition. To respond to sperm competition, rival males increase the transfer of SFPs during mating (Wigby et al. 2009; Hopkins et al. 2019). No‐rival males could be marginally more attractive to females if they have remained in better condition, as they avoid physical damage caused by aggressive interactions with rival males (Davis et al. 2018). Thus, via such damage, the perception of quality in rival males, or their ability to court females, may be compromised and they could suffer reduced mating success (Chen et al. 2002; Davis et al. 2018). Alternatively, no‐rival males could also secure more matings due to their ability to outcompete rival males. There are three possible explanations. First, territorial aggressive behavior can occur between rival males in close proximity (Chen et al. 2002). This would be detrimental to their ability to compete for mates as they would have less energy than the no‐rival mate to successfully court the female. It would be interesting in the future to test the extent that aggression between males in the rivals treatment vials underlies their subsequent mating phenotypes. Second, rival males may be less willing to court due to the perception of high competition (Weir et al. 2011). Courtship behavior is energetically costly (Cordts and Partridge 1996; Bretman et al. 2013) and rival males may benefit from withholding courtship until competition is reduced. Finally, it might be that rival males are habituated to the presence of other flies and do not respond as quickly in comparison to socially isolated males.

### INTERACTING EFFECTS OF PLASTICITY AND SEXUAL CONFLICT

Under some circumstances, sexual conflict is predicted to drive interacting plasticity to reach a fitness optimum between the interests of the male and the female, although the extent to which this occurs will depend upon the strength of sex‐specific selection and whether either sex has the upper hand in any conflict (Moore and Pizzari 2005; Yamaguchi and Iwasa 2015; McLeod and Day 2017; Day and McLeod 2018). The results here suggest that the expression of some reproductive traits (mating latency) is determined by interactions between the plastic responses of each sex, and others (mating duration, fecundity) by only one sex. Whether reproductive traits determined solely by the effects of the plasticity expressed by one sex reflect a resolution of sexual conflict over reproductive investment decisions (Yamaguchi and Iwasa 2015; McLeod and Day 2017; Day and McLeod 2018) is not yet clear and deserves further study.

Males that perceived themselves to be at high risk of sperm competition mated for significantly longer with females from all social environments. It has previously been observed that longer matings under these circumstances can transfer more cost‐inducing SFPs (Wigby et al. 2009). Thus, the heightened SFP allocation by such males should be evident in plastic responses in females to resist SFP effects. This could be evident as reduced willingness to mate with such males (slow mating latency or lower mating success) potentially modified by the female's own information on the likelihood of meeting any additional males (as signaled by their premating and/or mating social environment). The results are generally in line with this expectation. Specifically, in the choice scenarios, rival males were generally less successful at mating than were no‐rival males and there were significant interactions of mating latency with female social status. Rival treatment males were significantly slower to mate with same‐sex females under no choice, but significantly faster under choice conditions. The data suggest that females may be able to assess their own social environment and respond in a manner that mitigates SFP effects. Thus, selection may have favored males that can increase their mating propensity while also favoring females that can effectively assess their environment.

## Conclusion

We provide experimental evidence for the interacting effects of plasticity expressed by males and females. These plastic responses, although induced to increase the fitness interests of each sex, interact in the case of mating latency, but not in the case of mating duration and fecundity. The variable pattern of traits influenced by both sexes or by one is only partly consistent with theory and may reflect the outcome of sexual conflict (Moore and Pizzari 2005; Yamaguchi and Iwasa 2015; McLeod and Day 2017; Day and McLeod 2018). Future studies of reproductive behavior should carefully consider the sociosexual environment of both males and females.

## AUTHOR CONTRIBUTIONS

EF, AB, and TC devised the experiments. EF and SL conducted the research and collected and analyzed the data. EF, SL, and TC wrote this article. All authors contributed to the final draft.

## CONFLICT OF INTERESTS

Tracey Chapman is the Editor‐in‐Chief of Evolution and is a co‐author of this article. She was excluded from editorial decision‐making related to the acceptance and publication of this article. Editorial decision‐making was handled independently by Handling Editor Andrew McAdam to minimize bias. All other authors declare no conflict of interest.

## DATA ARCHIVING

The raw data are deposited in the DRYAD data depository (https://doi.org/10.5061/dryad.3tx95X6jc).

Associate Editor: Dr. T. Connallon

Handling Editor: Dr. A. G. McAdam

## Supporting information


**Table S1**: Summary statistics (mean, standard deviation (SD), 95% confidence interval (CI)) and sample sizes for all treatments across all experiments.
**Table S2**: Number of matings by male social environment in the choice experiment. Shown are the results of a Chi‐squared analysis of male mating success by male social treatment (rival or no‐rival).
**Table S3**: Model parameter estimates and test statistics for the effect of female social treatment on mating success, mating latency, mating duration and egg count in the choice scenario.
**Table S4**: Number of matings by male wing marker colour in the choice experiment. Shown are the results of a Chi‐squared analysis of male mating success by male wing colour (black or red).
**Table S5**: Model parameter estimates and test statistics for the effect of male and female social treatment on mating latency, mating duration and egg count in the no‐choice scenario.
**Table S6**: Model parameter estimates and test statistics for the effect of male social treatment on egg count within the different female social environments. Summaries are shown for the choice and no‐choice assays.
**Figure S1**. Kaplan‐Meier curves of the proportion of females mated over time from across the choice (panels A, C & E) experiment and no‐choice (panels B, D & F). Panels A & B show data for “alone” female treatment, panels C & D show “same‐sex” female treatment and panels E & F show mixed‐sex female treatment. Within each plot, data is split by male treatment (red lines: no‐rival treatment; blue lines: rival treatment).
