# Supplementary material for: Plastic responses of males and females interact to determine mating behavior
Source: Evolution. 2022 Aug 12;76(9):2116–29. doi: 10.1111/evo.14568 (PMC9544784; doi:10.1111/evo.14568)
Supplement: Supplementary file 1 — Table S1: Summary statistics (mean, standard deviation (SD), 95% confidence interval (CI)) and sample sizes for all treatments across all experiments. Table S2: Number of matings by male social environment in the choice experiment. Shown are the results of a Chi‐squared analysis of male mating success by male social treatment (rival or no‐rival). Table S3: Model parameter estimates and test statistics for the effect of female social treatment on mating success, mating latency, mating duration and egg count in the choice scenario. Table S4: Number of matings by male wing marker colour in the choice experiment. Shown are the results of a Chi‐squared analysis of male mating success by male wing colour (black or red). Table S5: Model parameter estimates and test statistics for the effect of male and female social treatment on mating latency, mating duration and egg count in the no‐choice scenario. Table S6: Model parameter estimates and test statistics for the effect of male social treatment on egg count within the different female social environments. Summaries are shown for the choice and no‐choice assays. Figure S1. Kaplan‐Meier curves of the proportion of females mated over time from across the choice (panels A, C & E) experiment and no‐choice (panels B, D & F). Panels A & B show data for “alone” female treatment, panels C & D show “same‐sex” female treatment and panels E & F show mixed‐sex female treatment. Within each plot, data is split by male treatment (red lines: no‐rival treatment; blue lines: rival treatment). [file EVO-76-2116-s001.pdf]

Plastic responses of males and females interact to determine mating behaviour

Fowler, E.K.[1*], Leigh, S.[1*], Bretman, A.[2], Chapman, T.[1#]

[1]School of Biological Sciences, University of East Anglia, Norwich Research Park, Norwich, NR4 7TJ, UK.

[2]School of Biology, Faculty of Biological Sciences, University of Leeds, Leeds, LS2 9JT, UK.

*joint first authors

Phone: + 44 (0)1603 5932107

[#]Author for correspondence: Tracey Chapman

e-mail: tracey.chapman@uea.ac.uk

Running head: Effects of behavioural plasticity in fruitflies

Keywords: phenotypic plasticity, cues, oviposition, mating duration, *Drosophila*

Supplementary Tables

Table S1: Summary statistics (mean, standard deviation (SD), 95% confidence interval (CI)) and sample sizes for all treatments across all experiments.

Table S2: Number of matings by male social environment in the choice experiment. Shown are the results of a Chi-squared analysis of male mating success by male social treatment (rival or no-rival).

Table S3: Model parameter estimates and test statistics for the effect of female social treatment on mating success, mating latency, mating duration and egg count in the choice scenario.

Table S4: Number of matings by male wing marker colour in the choice experiment. Shown are the results of a Chi-squared analysis of male mating success by male wing colour (black or red).

Table S5: Model parameter estimates and test statistics for the effect of male and female social treatment on mating latency, mating duration and egg count in the no-choice scenario.

Table S6: Model parameter estimates and test statistics for the effect of male social treatment on egg count within the different female social environments. Summaries are shown for the choice and no-choice assays.

Table S1: Summary statistics (mean, standard deviation (SD), 95% confidence interval (CI)) and sample sizes for all treatments across both experiments.

Variable	Female treatment	Male treatment	Sample size	Mean ± SD	CI (95%)
**Choice experiment**					
Proportion of matings secured	Alone	No rival	46	-	-
		Rival	46	-	-
	same-sex	No rival	42	-	-
		Rival	42	-	-
	mixed-sex	No rival	43	-	-
		Rival	43	-	-
Mating latency	Alone	No rival	25	3.72 ± 3.10 min	1.28
		Rival	21	4.14 ± 4.08 min	1.86
	same-sex	No rival	24	4.25 ± 3.64 min	1.54
		Rival	18	2.56 ± 1.15 min	0.57
	mixed-sex	No rival	24	4.33 ± 3.13 min	1.32
		Rival	18	4.56 ± 2.57 min	1.28
Mating duration	Alone	No rival	25	15.72 ± 2.15 min	0.89
		Rival	21	17.95 ± 3.92 min	1.78
	same-sex	No rival	24	16.67 ± 5.24 min	2.21
		Rival	18	16.83 ± 2.73 min	1.36
	mixed-sex	No rival	24	14.75 ± 2.40 min	1.01
		Rival	19	17.26 ± 3.54 min	1.71
Egg count	Alone	No rival	25	22.36 ± 14.59	6.02
		Rival	21	30.10 ± 20.23	9.21
	same-sex	No rival	24	28.08 ± 17.31	7.31
		Rival	17	24.24 ± 13.47	6.92
	mixed-sex	No rival	24	19.29 ± 11.46	4.84
		Rival	19	17.95 ± 8.44	4.07
**No choice experiment**					
Mating latency	Alone	No rival	46	12.74 ± 14.23 min	4.22
		Rival	44	12.18 ± 14.48 min	4.40
	same-sex	No rival	44	7.00 ± 5.80 min	1.76
		Rival	37	12.19 ± 13.63 min	4.55
	mixed-sex	No rival	43	13.88 ± 17.05 min	5.25
		Rival	43	14.65 ± 14.89 min	4.58
Mating duration	Alone	No rival	46	16.63 ± 2.64 min	0.79
		Rival	43	18.95 ± 4.45 min	1.37
	same-sex	No rival	44	17.36 ± 3.74 min	1.14
		Rival	37	19.41 ± 3.83 min	1.28
	mixed-sex	No rival	42	17.48 ± 2.96 min	0.92
		Rival	43	19.84 ± 4.60 min	1.41
Egg count	Alone	No rival	46	26.28 ± 17.28	5.13
		Rival	44	27.64 ± 17.64	5.36
	same-sex	No rival	44	22.07 ± 16.28	4.95
		Rival	36	30.28 ± 14.85	5.02
	mixed-sex	No rival	43	23.07 ± 18.11	5.57
		Rival	40	22.43 ± 13.86	4.43

Table S2: Number of matings by male social environment in the choice experiment. Shown are the results of a Chi-squared analysis of male mating success by male social treatment (rival or no-rival).

	**Female social treatment**									combined		
	alone			same-sex			mixed-sex					
**male social**	success	fail	χ^2	success	fail	χ^2	success	fail	χ^2	success	fail	χ^2
rival	21	25	$\chi^2 = 0.4$ *df* = 1 *p* = 0.53	18	24	$\chi^2 =$ 1.2 *df* = 1 *p* = 0.28	19	24	$\chi^2 = 0.7$ *df* = 1 *p* = 0.39	73	58	$\chi^2 = 3.0$ *df* = 1 *p* = 0.08
no-rival	25	21		24	18		24	19		58	73	

Table S3 Model parameter estimates and test statistics for the effect of male and female social treatment on mating success, mating latency, mating duration and egg count in the choice scenario. Female treatment abbreviations: same-sex (SS) and mixed-sex (MS).

Mating success

	Estimate	Std. Error	z-value	p-value
**Full model**				
(intercept)	-0.174	0.296	-0.589	0.556
Female treatment (SS)	-0.113	0.430	-0.264	0.792
Female treatment (MS)	-0.059	0.427	-0.139	0.889

Mating latency

	coef	exp(coef)	se(coef)	z	Pr(>|z|)
**Full model**					
Female treatment (SS)	-0.212	0.809	0.288	-0.736	0.462
Female treatment (MS)	-0.252	0.778	0.287	-0.877	0.380
Male treatment (rival)	-0.185	0.831	0.302	-0.613	0.540
Female treatment (SS):male treatment (rival)	0.100	2.718	0.441	2.268	0.023 *
Female treatment (MS):male treatment (rival)	-0.007	0.993	0.435	-0.017	0.986

Mating duration

	Estimate	Std. Error	t-value	p-value
**Full model**				
(intercept)	15.720	0.701	22.437	<0.001
Male treatment (rival)	2.232	1.037	2.153	0.033 *
Female treatment (SS)	0.947	1.001	0.946	0.346
Female treatment (MS)	-0.970	1.001	-0.969	0.335
Male treatment (rival):female treatment (SS)	-2.066	1.506	-1.372	0.173
Male treatment (rival):female treatment (MS)	0.281	1.494	0.188	0.851
**Reduced model 1**				
(intercept)	15.978	0.590	27.080	<0.001
Male treatment (rival)	1.667	0.618	2.696	0.008 **
Female treatment (SS)	0.046	0.750	0.061	0.952
Female treatment (MS)	-0.854	0.745	-1.146	0.254

Comparison of fit

Model	Resid. Df	RSS	Df	SS	F	Pr(>F)
Full	125	1534.0				
Reduced 1	127	1568.4	2	34.394	1.401	0.250

	Estimate	Std. Error	t-value	p-value
**Final reduced model**				
(intercept)	15.712	0.411	38.231	<0.001
Male treatment (rival)	1.667	0.618	2.699	0.008 **

Comparison of fit

Model	Resid. Df	RSS	Df	SS	F	Pr(>F)
Reduced 1	127	1568.4				
Final reduced	129	1590.6	2	22.21	0.899	0.507

Post-mating eggs

	Estimate	Std. Error	z-value	p-value
**Full model**				
(intercept)	3.107	0.126	24.737	<0.001
Female treatment (SS)	0.228	0.170	1.341	0.182
Female treatment (MS)	-0.148	0.187	-0.791	0.431
Male treatment (rival)	0.297	0.172	1.723	0.087
Female treatment (SS):male treatment (rival)	-0.445	0.253	-1.754	0.082
Female treatment (MS):male treatment (rival)	-0.369	0.273	-1.352	0.179
**Reduced model 1**				
(intercept)	3.232	0.101	31.932	<0.001
Female treatment (SS)	0.025	0.127	0.196	0.845
Female treatment (MS)	-0.325	0.138	-2.361	0.020 *
Male treatment (rival)	0.047	0.109	0.431	0.667

Comparison of fit

Model	Resid. Df	Resid. Dev	Df	Deviance	F	P(>|Chi|)
Full	124	1394.8				
Reduced model 1	126	1426.0	2	31.204	1.769	0.175

	Estimate	Std. Error	z-value	p-value
**Final reduced model**				
(intercept)	3.254	0.087	37.377	<0.001
Female treatment (SS)	0.023	0.126	0.181	0.857
Female treatment (MS)	-0.326	0.137	-2.374	0.019 *

Comparison of fit

Model	Resid. Df	Resid. Dev	Df	Deviance	F	P(>|Chi|)
Reduced model 1	126	1426.0				
Final reduced model	127	1427.7	1	1.683	0.185	0.668

Table S4: Number of matings by male wing marker colour in the choice experiment. Shown are the results of a Chi-squared analysis of male mating success by male wing colour (black or red).

	**Female social treatment**											
	alone			same-sex			mixed-sex			combined		
**male wing colour**	success	fail	χ^2	success	fail	χ^2	success	fail	χ^2	success	fail	χ^2
black	26	20	χ^2 = 1.1 *df* = 1 *p* = 0.30	21	21	χ^2 = 0 *df* = 1 *p* = 1	25	18	χ^2 = 1.7 *df* = 1 *p* = 0.20	72	59	χ^2 = 2.2 *df* = 1 *p* = 0.14
red	20	26		21	21		18	25		59	72	

Table S5 Model parameter estimates and test statistics for the effect of male and female social treatment on mating latency, mating duration and egg count in the no-choice scenario. Female treatment abbreviations: same-sex (SS) and mixed-sex (MS).

Mating latency

	coef	exp(coef)	se(coef)	z	Pr(>|z|)
**Full model**					
Male treatment (rival)	-0.039	0.961	0.211	-0.186	0.852
Female treatment (SS)	0.497	1.644	0.212	2.341	0.019 *
Female treatment (MS)	-0.330	0.719	0.214	-1.541	0.123
Female treatment (SS):male treatment (rival)	-0.631	0.532	0.309	-2.045	0.041 *
Female treatment (MS):male treatment (rival)	0.057	1.058	0.303	0.188	0.851

Mating duration

	Estimate	Std. Error	t-value	p-value
**Full model**				
(intercept)	16.630	0.556	29.991	<0.001
Male treatment (rival)	2.323	0.798	2.912	0.004 **
Female treatment (SS)	0.733	0.793	0.925	0.356
Female treatment (MS)	0.846	0.803	1.054	0.293
Male treatment (rival):female treatment (SS)	-0.281	1.158	-0.243	0.808
Male treatment (rival):female treatment (MS)	0.038	1.141	0.033	0.973

**Reduced model 1**						
(intercept)		16.667	0.458		36.435	<0.001
Male treatment (rival)		2.247	0.470		4.781	<0.001
Female treatment (SS)		0.603	0.576		1.047	0.296
Female treatment (MS)		0.867	0.569		1.525	0.129
**Comparison of fit**						
Model	Resid. Df	RSS	Df	SS	F	Pr(>F)
Full	249	3522.1				
Reduced 1	251	3523.3	2	1.251	0.044	0.957
		Estimate	Std. Error		t-value	p-value
**Final reduced model**						
(intercept)		17.144	0.326		52.526	<0.001
Male treatment (rival)		2.254	0.470		4.797	<0.001
**Comparison of fit**						
Model	Resid. Df	RSS	Df	SS	F	Pr(>F)
Reduced 1	251	3523.3				
Final reduced	253	3557.7	2	34.4	1.227	0.295
**Post-mating eggs**						
		Estimate	Std. Error		z-value	p-value
**Full model**						
(intercept)		3.269	0.095		34.388	<0.001
Male treatment (rival)		0.050	0.134		0.374	0.709
Female treatment (SS)		-0.175	0.142		-1.227	0.221
Female treatment (MS)		-0.130	0.142		-0.921	0.358
Female treatment (SS):male treatment (rival)		0.266	0.198		1.342	0.181
Female treatment (MS):male treatment (rival)		-0.079	0.203		-0.387	0.699
**Reduced model**						
(intercept)		3.237	0.080		40.579	<0.001
Male treatment (rival)		0.113	0.083		1.361	0.175
Female treatment (SS)		-0.040	0.099		-0.408	0.684
Female treatment (MS)		-0.168	0.102		-1.654	0.100
**Comparison of fit**						
Model	Resid. Df	Resid. Dev	Df	Deviance	F	P(>|Chi|)

Full	247	3400.3				
Reduced model	249	3433.4	2	33.081	1.514	0.222

Table S6. Model parameter estimates and test statistics for the effect of male social treatment on egg count within the different female social environments. Summaries are shown for the choice and no-choice assays.

**Post-mating eggs, choice**				
	Estimate	Std. Error	t-value	p-value
**Alone females**				
(intercept)	3.107	0.143	21.785	< 0.001
Male treatment (rival)	0.297	0.196	1.517	0.136
**Same-sex females**				
(intercept)	3.335	0.118	28.30	< 0.001
Male treatment (rival)	-0.147	0.191	-0.77	0.446
**Mixed-sex females**				
(intercept)	2.960	0.110	26.995	< 0.001
Male treatment (rival)	-0.072	0.168	-0.429	0.67

**Post-mating eggs, no-choice**				
	Estimate	Std. Error	t-value	p-value
**Alone females**				
(intercept)	3.269	0.097	33.794	< 0.001
Male treatment (rival)	0.050	0.137	0.368	0.714
**Same-sex females**				
(intercept)	3.094	0.101	30.656	< 0.001
Male treatment (rival)	0.316	0.139	2.279	0.025 *
**Mixed-sex females**				
(intercept)	3.139	0.108	29.152	< 0.001
Male treatment (rival)	-0.028	0.156	-0.181	0.856

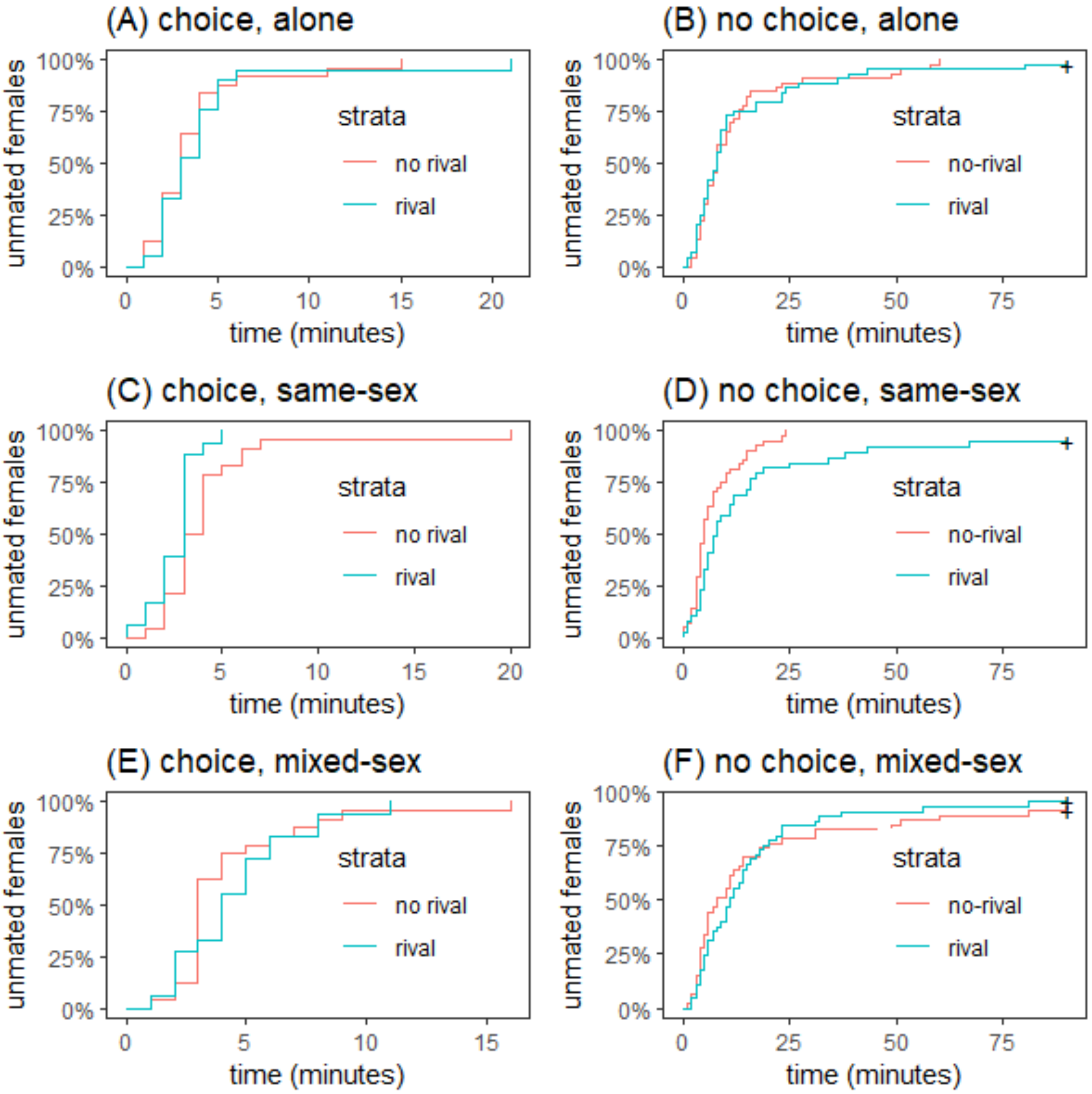

Figure S1. Kaplan-Meier curves of the proportion of females mated over time from across the choice (panels A, C & E) experiment and no-choice (panels B, D & F). Panels A & B show data for "alone" female treatment, panels C & D show "same-sex" female treatment and panels E & F show mixed-sex female treatment. Within each plot, data is split by male treatment (red lines: no-rival treatment; blue lines: rival treatment).
